# SAR Target Recognition via Meta-Learning and Amortized Variational Inference

**DOI:** 10.3390/s20205966

**Published:** 2020-10-21

**Authors:** Ke Wang, Gong Zhang

**Affiliations:** 1 School of Electronic and Information Engineering, Nanjing University of Aeronautics and Astronautics, Nanjing 211100, China; wangke_81x@163.com; 2 Key Laboratory of Radar Imaging and Microwave Photonics, Ministry of Education, Nanjing University of Aeronautics and Astronautics, Nanjing 211100, China

**Keywords:** automatic target recognition, meta-learning, amortized variational inference

## Abstract

The challenge of small data has emerged in synthetic aperture radar automatic target recognition (SAR-ATR) problems. Most SAR-ATR methods are data-driven and require a lot of training data that are expensive to collect. To address this challenge, we propose a recognition model that incorporates meta-learning and amortized variational inference (AVI). Specifically, the model consists of global parameters and task-specific parameters. The global parameters, trained by meta-learning, construct a common feature extractor shared between all recognition tasks. The task-specific parameters, modeled by probability distributions, can adapt to new tasks with a small amount of training data. To reduce the computation and storage cost, the task-specific parameters are inferred by AVI implemented with set-to-set functions. Extensive experiments were conducted on a real SAR dataset to evaluate the effectiveness of the model. The results of the proposed approach compared with those of the latest SAR-ATR methods show the superior performance of our model, especially on recognition tasks with limited data.

## 1. Introduction

Synthetic aperture radar (SAR) is an active remote sensor with all day and night, high-resolution, and wide-area imaging capabilities. Because of these unique capabilities, SAR is widely used in geoscience and remote sensing. Today, numerous SAR sensors are operating on spaceborne and airborne platforms and are imaging ground targets for surveillance and reconnaissance. For efficient interpretation of SAR image data, SAR automatic target recognition (SAR-ATR) system are being developed. SAR-ATR aims to detect and recognize targets, such as trucks and armored personnel carriers, in SAR images. The workflow of an end-to-end SAR-ATR system includes three stages: detection, low-level classification, and high-level classification [1]. Once an SAR image enters the system, detectors, such as constant false-alarm rate (CFAR) detectors, locate candidate targets in the images [2]. The region of interest (ROI), consisting of the true target and background clutter, is extracted around each candidate target. The clutter is then analyzed and filtered out in the low-level classification. Finally, the class or even the model of the target is identified in the high-level classification. In this paper, we focus on the third stage, that is, high-level classification.

Traditional SAR-ATR methods are generally classified into two categories: feature-based and model-based. Feature-based methods extract discriminative features from SAR images and train the classifiers with these features. The features can be extracted in the spatial domain, such as templates, each of which is an average representation of a target at a particular azimuth angle [3]. Feature extraction can also be carried out in the transformation domain, where the images are transformed into low-dimensional features. There exist various transformations, such as kernel principal component analysis (KPCA) [4], structure-preserving projection [5], and locality discriminant projection [6]. Sparse and redundant representation techniques were developed and subsequently introduced to the field of SAR-ATR. Sun et al. [7] extracted scale-invariant features and pixel amplitudes from images and then used the joint dynamic sparse representation classification technique to classify them. Dong et al. combined the monogenic signal with multiple classification methods, such as sparse representation classification [8], manifold learning [9], and multi-task learning [10].

Unlike feature-based methods, model-based approaches employ computer-aided design (CAD) modeling and electromagnetic computing to provide physical descriptions of targets. Target characteristics, which are regions or parameters of scattering centers, are used as references for recognition. Zhou et al. [11] used wideband measurements to establish the targets’ 3-D global scattering centers. These scattering centers were projected onto a 2-D imaging plane and were compared to the test images. In [12,13], researchers first constructed accurate CAD models of targets. The scattering centers of the targets were generated by running an electromagnetic simulator on the targets’ CAD models. Ding et al. [14] proposed a region matching metric suitable for 3-D scattering center models. They represented scattering centers as binary regions and developed a coarse-to-fine region matching algorithm to measure the distance between two scattering centers.

Both feature-based and model-based methods require experts to manually design features or models suitable for SAR images. Convolutional neural networks (CNNs) [15] have received much attention in SAR-ATR due to their capacity to automatically learn hierarchical image features. CNN generally consists of multiple convolutional layers used for feature extraction and fully connected (FC) layers used for feature classification. To reduce the number of network parameters, Chen et al. [16] built an all-convolutional network without FC layers. Wagner [17] replaced the last FC layer in the network with a support vector machine (SVM), which increased training complexity but boosted recognition performance. Min et al. [18] used the student-teacher paradigm to compress a deep CNN into a micro CNN (MCNN) containing only two layers. Speckle noise, caused by the unique SAR imaging mechanism, degrades the performance of SAR-ATR. To reduce the influence of speckle noise, Cho et al. [19] proposed a multiple feature-based CNN (MFCNN) that uses max-pooling and average-pooling in parallel to aggregate features. Kwak et al. [20] added regularization to the training process of CNN to minimize feature variations caused by speckle noise. The trained CNN extracts noise-robust features and hence has improved recognition performance.

Although the CNN achieves state-of-the-art recognition performance, it requires collecting and annotating huge amounts of training data. Collecting SAR data, however, is limited by cost and security considerations. Data augmentation is a common practice to overcome this limitation. Ding et al. [21] used augmentation operations, including translation, rotation, and the addition of noise, to generate synthetic SAR images. Jiang et al. [22] used Gabor filters to extract multi-scale and multi-directional features of SAR images. These features, which are more diverse than raw images, can be used as training samples for the CNN. Similarly, Pei et al. [23] trained a CNN with multiview SAR data, which is a combination of SAR images at different azimuth angles. The CNN extracted multiview image features and fused them in a parallel network. In addition to data augmentation, researchers have also utilized external data sources and developed corresponding transfer learning frameworks. Transfer learning acquires prior information from external sources such as optical data [24] and virtual SAR data [25]. It then uses this information to help train CNNs that recognize real SAR targets.

Recently, meta-learning methods [26,27] have made significant progress in few-shot image classification in which each class has few labeled samples available for training. By learning priors from many training tasks, meta-learning solves new testing tasks using only a few samples. Acquiring these priors requires the training and testing tasks to share some common structures, such as visual or semantic features. Tang et al. [28] proposed an inference model based on Siamese networks, which not only improves the accuracy of few-shot SAR recognition but also reduces the prediction time. Wang et al. [29] integrated model-agnostic meta-learning (MAML) with domain adaptation to solve cross-domain and cross-task SAR-ATR problems. In this paper, we solve SAR-ATR tasks with small data in a meta-learning framework. Simulated SAR data [30] are also introduced to compensate for the lack of real data. We build a meta-learning model consisting of global parameters and task-specific parameters. The model is meta-learned using sufficient simulated SAR data. After meta-learning, it retains the global parameters and uses real SAR data to update task-specific parameters. To reduce the model uncertainty caused by small training data, the model places probability distributions over task-specific parameters. These parameters, vast in number, are estimated by amortized variational inference (AVI) [31] to reduce the computation and storage cost. Most relevant to our method is the work of [32,33], in which amortized networks (e.g., plain neural networks) were used to approximate task-specific parameters. By contrast, our model uses variational inference to clarify the errors introduced by amortized approximation, thereby improving the training objective function. Furthermore, we propose a novel amortized network implemented with set-to-set functions [34] to boost the performance of AVI.

The contributions of this paper are summarized as follows:

(1) We propose a novel recognition model integrating meta-learning and AVI. The model can recognize new targets with a small amount of real data.

(2) To reduce the model uncertainty caused by small data, task-specific parameters of the model are modeled by probability distributions and are inferred by AVI.

(3) The amortized network of AVI is implemented with set-to-set functions, thereby improving its performance.

## 2. Methods

### 2.1. Model Framework

In this paper, SAR-ATR with small data is defined and solved in a probabilistic meta-learning framework. The task, a random combination of training samples, is the independent training unit of the model. Assume that we have a task set $\;{\left\{\tau_{i}\right\}}_{i=1}^{M}$ of *M* tasks, where the $i^{th}$ task $\tau_{i}={\left\{\left(x_{ij,}y_{ij}\right)\right\}}_{j=1}^{N}\;$ has *N* samples, each of which contains an image $x_{ij}\;$ and its ground-truth label $\;y_{ij}$. The samples of $\tau_{i}$ are then split into two disjoint parts: a support set $\tau_{i}^{s}=\;{\left\{\left(x_{ij,}y_{ij}\right)\right\}}_{j=1}^{L}$ for training and a query set $\;\tau_{i}^{q}=\;{\left\{\left(x_{ij,}y_{ij}\right)\right\}}_{j=L+1}^{N}$ to evaluate training effectiveness.

**Figure 1:**
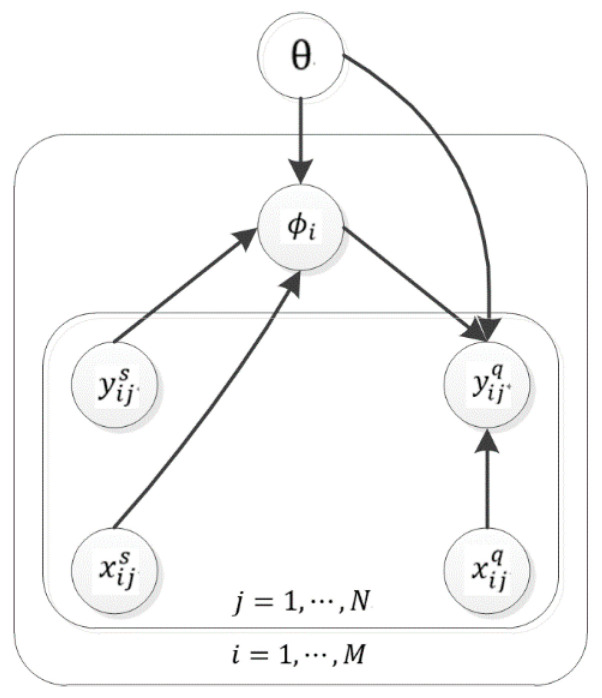
The graphical model for the meta-learning framework. Open circles represent one or a group of random variables. The arrows indicate probabilistic dependencies between random variables.

A probabilistic graph is employed to visually specify how components depend on each other in the model framework. Figure 1 shows that the framework consists of global parameters *θ*, task-specific parameters $\;\phi_{i}$, and samples that include support and query sets. Three principles guide the choice of model framework. First, we assume that all the tasks share a common structure parameterized by *θ*, which assists the model in solving new tasks with few training samples. Second, we employ the point estimate for *θ* because it is shared between tasks and its uncertainty decreases as the number of tasks increases. Third, the task-specific parameters $\;\phi_{i}$ are distributionally estimated to deal with the model uncertainty caused by small training data.

To simplify the symbols, the sample sets, $\left\{x_{ij}^{s}|\forall j\right\}$, $\;\left\{y_{ij}^{s}|\forall j\right\}$, $\;\left\{x_{ij}^{q}|\forall j\right\}$, and $\left\{y_{ij}^{q}|\forall j\right\}\;$ are abbreviated as $x_{i}^{s},\;y_{i}^{s},\;x_{i}^{q},\;\mathrm{and}\;y_{i}^{q}$, respectively. According to the maximum likelihood criterion, our goal is to train a model that utilizes the knowledge in $\;\left\{x_{i}^{s},y_{i}^{s}\right\}$ to predict the labels $y_{i}^{q}\;$ of new images $\;x_{i}^{q}$ accurately. The log-likelihood ℒ is defined as:

(1) $$ℒ=log\left[\overset{M}{\underset{i=1}{\prod }}p\left(y_{i}^{q}|x_{i}^{q},x_{i}^{s},y_{i}^{s},\theta \right)\right]\;=\overset{M}{\underset{i=1}{\sum }}log\left[\int p\left(y_{i}^{q},\phi_{i}|x_{i}^{q},x_{i}^{s},y_{i}^{s},\theta \right)d\phi_{i}\right],$$

where M  is the total number of tasks. As shown in Figure 1, $\;y_{i}^{q}$ is conditionally independent of $x_{i}^{s}$ and $y_{i}^{s}$, and $\phi_{i}$ is conditionally independent of $x_{i}^{q}.\;$According to the above dependency relationships, we factorize the joint distribution in (1) into two terms:

(2) $$ℒ=\overset{M}{\underset{i=1}{\sum }}log\left[\int p\left(y_{i}^{q}|x_{i}^{q},\phi_{i},\theta \right)p\left(\phi_{i}|x_{i}^{s},y_{i}^{s},\theta \right)d\phi_{i}\right],$$

where $\;p\left(\phi_{i}|x_{i}^{s},y_{i}^{s},\theta \right)$, the posterior distribution over $\phi_{i},\;$is learned by the support set $\;\left\{x_{i}^{s},y_{i}^{s}\right\}\;$. The predictive distribution $p\left(y_{i}^{q}|x_{i}^{q},\phi_{i},\theta \right)$ is inferred by query data $x_{i}^{q}$ and learned $\phi_{i}$. Computing (2)  requires integration over all the values of $\;\phi_{i}$, which is typically intractable for complex or large-scale models. Therefore, variational inference [35] is introduced to approximate the posterior $p\left(\phi_{i}|x_{i}^{s},y_{i}^{s},\theta \right)$. This involves a variational distribution $q\left(\phi_{i};\lambda_{i}\right)$, specified by a set of variational parameters $\;\lambda_{i}$. We rewrite the integral in (2) as expectations and use Jensen’s inequality to obtain its lower bound ${ℒ}_{VI}$:

(3) $${ℒ}_{VI}=\overset{M}{\underset{i=1}{\sum }}E_{q\left(\phi_{i};\lambda_{i}\right)}\left[log\left[p\left(y_{i}^{q}|x_{i}^{q},\phi_{i},\theta \right)\frac{p\left(\phi_{i}|x_{i}^{s},y_{i}^{s},\theta \right)}{q\left(\phi_{i};\lambda_{i}\right)}\right]\right]\;=\overset{M}{\underset{i=1}{\sum }}E_{q\left(\phi_{i};\lambda_{i}\right)}\left[log\left[p\left(y_{i}^{q}|x_{i}^{q},\phi_{i},\theta \right)\right]\right]-KL\left(q\left(\phi_{i};\lambda_{i}\right)||p\left(\phi_{i}|x_{i}^{s},y_{i}^{s},\theta \right)\right),$$

where the first term represents the predictive log-likelihood given a variational distribution $\;q\left(\phi_{i};\lambda_{i}\right)$. The second term is the Kullback-Leibler (KL) divergence between $q\left(\phi_{i};\lambda_{i}\right)$ and $\;p\left(\phi_{i}|x_{i}^{s},y_{i}^{s},\theta \right)$. Minimizing the KL divergence will bridge the gap between two distributions to eliminate approximation errors.

During meta-learning, large numbers of tasks are generated by randomly drawing training samples from the dataset. With the increasing number of tasks, learning variational parameters $\lambda_{i}\;$ for each $\phi_{i}$ is challenging due to the cost of storage and computing. Therefore, we introduce an AVI that combines an amortized inference network with variational inference. AVI takes a task $\tau_{i}\;$ as input and uses an inference network shared between all tasks to predict $\;\phi_{i}$. Thus, the optimization problem of (3) turns into:

(4) $${ℒ}_{VI}=\overset{M}{\underset{i=1}{\sum }}E_{q_{\varphi }\left(\phi_{i}|x_{i}^{s},y_{i}^{s},\theta \right)}\left[log\left[p\left(y_{i}^{q}|x_{i}^{q},\phi_{i},\theta \right)\right]\right]-KL\left(q_{\varphi }\left(\phi_{i}|x_{i}^{s},y_{i}^{s},\theta \right)||p\left(\phi_{i}|x_{i}^{s},y_{i}^{s},\theta \right)\right),$$

where $q_{\varphi }\left(\phi_{i}|,x_{i}^{s},y_{i}^{s},\theta \right)\;$ is the approximation of $q\left(\phi_{i};\lambda_{i}\right)$, and the inference network is parameterized by φ. After performing AVI, the model only needs to learn and store globally shared parameters (i.e., θ and  φ) throughout the meta-learning process, while $\phi_{i}$ is predicted by globally shared parameters and training samples. Finally, the model performs the following optimization:

(5) $$\underset{\theta ,\varphi }{\mathrm{argmin}}\overset{M}{\underset{i=1}{\sum }}-E_{q_{\varphi }\left(\phi_{i}|x_{i}^{s},y_{i}^{s},\theta \right)}\left[log\left[p\left(y_{i}^{q}|x_{i}^{q},\phi_{i},\theta \right)\right]\right]+\alpha \;\;\;\;\;\;\;\times KL\left(q_{\varphi }\left(\phi_{i}|x_{i}^{s},y_{i}^{s},\theta \right)||p\left(\phi_{i}|x_{i}^{s},y_{i}^{s},\theta \right)\right),$$

where the first term of (5) is the cross-entropy loss of the query set, while the KL term can be viewed as a training regularization step. Due to the varying range of values for the two terms, the weight coefficient α between them needs to be down-weighted. $q_{\varphi }\left(\phi_{i}|x_{i}^{s},y_{i}^{s},\theta \right)$ is a Gaussian distribution with mean and covariance determined by data $\left\{x_{i}^{s},y_{i}^{s}\right\}\;$ and parameters {θ,φ}. $p\left(\phi_{i}|x_{i}^{s},y_{i}^{s},\theta \right)$ is defined experimentally and also a Gaussian distribution. Its mean is identical to that of $q_{\varphi }\left(\phi_{i}|x_{i}^{s},y_{i}^{s},\theta \right)$, but its covariance is an identity matrix. For the detailed calculation method of KL divergence, one can refer to [36].

### 2.2. Model Structure

**Figure 2:**
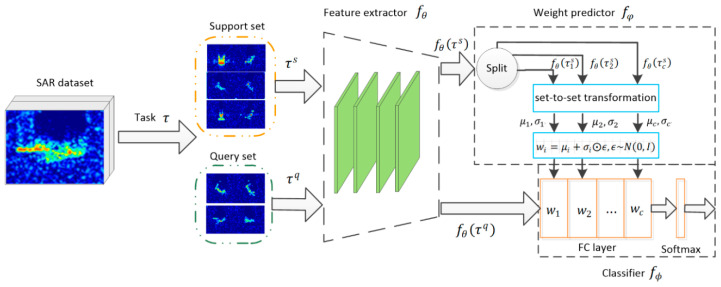
The overall structure of our model. The model samples a task from the synthetic aperture radar (SAR) dataset and divides it into a support set and a query set. The feature extractor uses a four-layer convolutional neural network (CNN) to extract image features. The classifier identifies the category of image features, and its weight is generated by the weight predictor.

As shown in Figure 2, the model is composed of three modules: a feature extractor $\;f_{\theta }$ with global parameters, a classifier $\;f_{\phi }$ with task-specific parameters, and a weight predictor $\;f_{\varphi }$. Once the images enter the model, their low-dimensional features are extracted by $f_{\theta }$. The weight predictor $f_{\varphi }\;$ then uses support set features $\;f_{\theta }\left(\tau^{s}\right)\;$ to predict the weights of $\;f_{\phi }$. Finally, the predictive distribution $p\left(y_{i}^{q}|x_{i}^{q},\phi_{i},\theta \right)$ is produced by $f_{\phi }$ using query set features $\;f_{\theta }\left(\tau^{q}\right)$.

**Figure 3:**
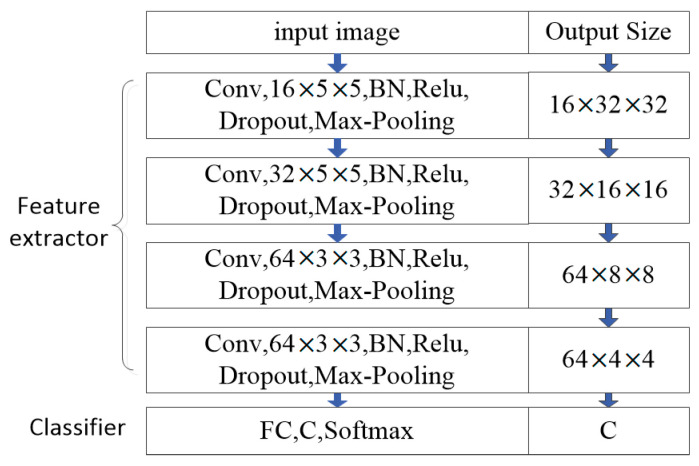
The network configuration of our model.

The detailed network configuration of the model is shown in Figure 3. The feature extractor uses a CNN that contains four convolutional blocks to map images into features. Each convolutional block sequentially performs convolution (Conv), batch normalization (BN), nonlinear activation, dropout, and max-pooling operations on the inputs. BN is essential in the network for accelerating the training convergence. Furthermore, it can effectively improve training stability, especially when training data are scarce. The classifier consists of an FC layer and a softmax layer, where *C* is the number of classes.

Before being sent to the feature extractor, the SAR images are first converted to a logarithmic scale, and their pixel values are normalized to [−1.0, 1.0]. The size of normalized images is then cropped to 64 × 64 to reduce the influence of clutter and noise. The first convolutional block convolves input images by 16 filters with a kernel size of 5 × 5. Its outputs are 16 feature maps of size 32 × 32 due to spatial zero paddings and a 2 × 2 max-pooling. The next three convolutional blocks perform similar processing on input feature maps. Finally, the feature extractor outputs 64 feature maps of the size 4 × 4 and flattens them into a 1024-D feature vector. The classifier determines the class to which the feature vector belongs. These feature vectors are also used to learn the weights of the classifier. The weight predictor uses set-to-set functions to transform feature vectors, extracts distribution parameters (e.g., mean and variance), and samples the classifier weights by these parameters.

### 2.3. Weight Predictor

Predicting task-specific weights for the classifier is challenging since its training samples per task are limited. It has been observed that in the last FC layer of a CNN, the weight vector and the input feature vector are highly similar in structure. Previous works in [32,33] regarded the mean of image features in one class as a class proxy. They inputted the proxy into amortized networks to predict classifier weights for this class. This idea is too rigid for cases in which the image features follow a complex distribution containing multiple cluster centers. Our model uses feature sets instead of feature means to infer classifier weights. The feature sets are first transformed by set-to-set functions taking into account both the inter-class and the intra-class information. Next, the model uses the transformed features to infer the distribution of classifier weights.

**Figure 4:**
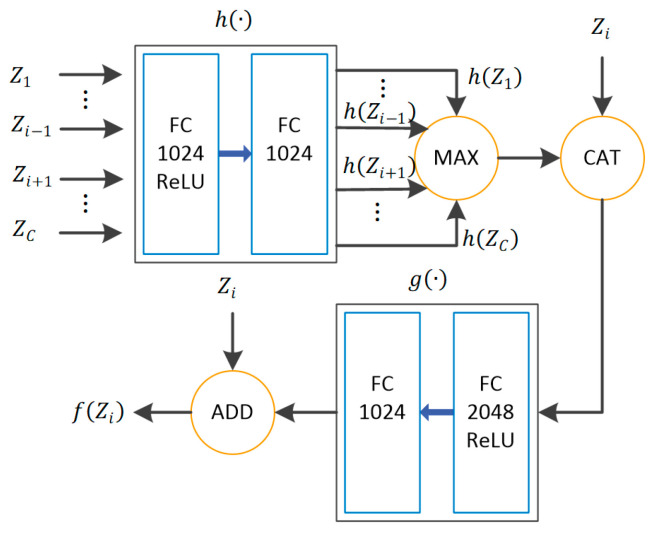
Illustration of set-to-set transformation. MAX denotes the max-pooling operation, CAT denotes vector concatenation, and ADD denotes vector addition.

Given a feature set ${\left\{Z_{i}\right\}}_{i=1}^{C}$ of *C* classes, where $Z_{i}$ represents image features belonging to class  i. The set-to-set transformation function $f\left(Z_{i}\right)\;$ is defined as

(6) $$f\left(Z_{i}\right)=Z_{i}+g\left(Z_{i}⊕\underset{j\neq i}{\max}h\left(Z_{j}\right)\right),$$

where ⊕  denotes vector concatenation, and g(·) and h(·) are nonlinear mappings. We use cascaded FC layers with ReLU activation to implement these nonlinear mappings. Figure 4 shows the transformation process of feature sets. For each $Z_{i}$, its complement elements $\left\{Z_{j},j\neq i\right\}$ are first transformed into some representations $\left\{h\left(Z_{j}\right),j\neq i\right\}\;$ by nonlinear mappings. The representations are aggregated as context data (inter-class information) by a maximum operator. Next, we concatenate $Z_{i}$ with the context vector and input it to the FC layers to obtain the residual mapping $\;g\left(Z_{i}\right)$.

$g\left(Z_{i}\right)\;$ can also be regarded as a conditioned mapping that considers other classes in the set. Finally, we add $Z_{i}$ and $g\left(Z_{i}\right)\;$ to obtain $f\left(Z_{i}\right).\;$ As stated in [34], the maximum operator in Figure 4 can be replaced by a sum operator. However, we experimentally found that the maximum operator performs better than the sum operator.

To reduce the model uncertainty caused by small data, the classifier weights are random variables that are inferred by AVI. In practice, the weights are task-specific parameters $\;\phi_{i}$, where *i* is the index of the tasks. We formulate $\;\phi_{i}\;$ as a stochastic matrix; thus, $\;\phi_{i}=\left[w_{1},\cdots ,w_{j},\cdots ,w_{C}\right]\in R^{D\times C}$, where *C* is the number of classes and *D* is the dimension of feature vectors. The distribution of $w_{j}\;$ is specified as a factorized Gaussian distribution $N\left(w_{j}|\mu_{j},diag\left(\sigma_{j}^{2}\right)\right)$, where $\mu_{j}$ and $diag\left(\sigma_{j}^{2}\right)$ are the mean vector and diagonal covariance matrix of $w_{j}$, respectively. The weight predictor $f_{\varphi }\;$ uses set-to-set functions to transform support set features $\;f_{\theta }\left(\tau^{s}\right)$, produces parameters $\;\mu_{j}$ and $\sigma_{j}^{2}$ for each $w_{j}$, and samples $w_{j}$ with these parameters. To facilitate the backpropagation of gradients, $f_{\varphi }$ samples $\;w_{j}$ with a local reparameterization trick instead of sampling $\;w_{j}$ directly. The sampling of $w_{j}\;$ is defined as follows:

(7) $$w_{j}\sim N\left(w_{j}|\mu_{j},diag\left(\sigma_{j}^{2}\right)\right)↔w_{j}=\mu_{j}+\sigma_{j}⊙\epsilon ,\;\epsilon \sim N\left(0,I\right)\;,$$

where $w_{j}$ is represented by a linear function of Gaussian variables ϵ, and ⊙  denotes the element-wise product.

## 3. Results and Discussion

### 3.1. Training Details

The workflow of our model includes three stages: meta-learning, updating, and testing. During meta-learning, the model learns parameters θ and φ using simulated SAR data. In the updating stage, the model freezes θ and uses a small amount of real SAR data to update  φ. The remaining real data are used to test the model. To mimic the meta-learning scenario, both real and simulated data are organized as *N*-way, *K*-shot classification tasks. To construct a task with support and query splits, we randomly chose *N* classes from the dataset and then collected (*K* + *L*) images from each class. These images were then divided into two disjoint subsets: a support set of size *N* × *K* and a query set of size *N* × *L*. We used *N* = 10, *K* = 5, and *L* = 15 in all the tests. Our model was trained by the ADAM optimizer [37] with a learning rate of 0.001. The regularization term (the KL divergence of Equation (5)) and dropout operation are only used in the meta-learning stage. The weight coefficient α and the drop rate are set to 0.0001 and 0.5, respectively. All the experiments were carried out on a computer configured with Intel i5-8400 CPU, GeForce GTX 1080Ti GPU and 16 GB RAM. The model needed to be trained for 14,000 iterations in the meta-learning stage and 300 iterations in the updating phase, and each iteration took 0.19 s. In the testing stage, it took 0.002 s for the model to recognize each target image.

### 3.2. Datasets

The model was trained on simulated SAR data and then updated and tested on real SAR data. The real SAR data were collected by an SAR system operating in X-band (9.6 GHz), HH-polarization, and spotlight mode, in support of the Moving and Stationary Target Acquisition and Recognition (MSTAR) project. The target images were imaged over a 360° azimuth angle and had a spatial resolution of 0.3 × 0.3 m. A huge number of ground target images with various classes, azimuth angles, depression angles, and so on, were gathered. Some of these images are publicly available and are widely used as a benchmark for SAR-ATR testing [38]. Table 1 summarizes the publicly released MSTAR dataset, including ten classes, each with hundreds of images.

The simulated dataset was generated and shared by Kusk et al. [30] at the Technical University of Denmark. The target’s radar cross-section (RCS) was generated by an electromagnetic computing software that takes the target’s CAD model as input. The RCS was then passed to a postprocessing tool that models thermal noise, terrain clutter, and SAR focusing, to produce simulated SAR images. The detailed simulation parameters are listed in Table 2.

The simulated dataset includes seven vehicles: bulldozer, bus, car, hummer, motorbike, tank, and truck. Each vehicle contains two variants built by different CAD models. In our experimental setup, each variant was viewed as an independent class, thereby establishing a dataset containing images of fourteen types of targets. The size and resolution of the simulated images are consistent with those of the real images, but the imaging angles are more diverse. The simulated images were acquired at azimuth angles from 0° to 360° at 5° intervals and a few depression angles (15°, 17°, 25°, 30°, 35°, 40°, and 45°). Table 3 lists the name, the CAD model and the number of images per class in the simulated dataset. In each class, the number of images is the product of the azimuths and the depressions.

### 3.3. Reference Methods

To quantitatively evaluate the model, we employed several state-of-the-art recognition methods as references, which are summarized in Table 4. Among these methods, class-dependent structure preserving projection (CDSPP) and kernel robust locality discriminant projection (KRLDP) are based on discriminant projection, kernel sparse representation (KSR) and tri-task joint sparse representation (TJSR) are based on sparse representation, and the rest are deep learning methods. The experimental results of CNN, transfer learning (TFL), probabilistic meta-learning (PML), MobileNet, and predicting parameters from activations (PPA) were obtained from our implementations. Their network structures are similar to that of our model. The results of other methods in Table 4 are cited directly from their papers.

### 3.4. Results under Standard Operation Conditions

Operation conditions (OCs), the working environment in which SAR sensors acquire images, have a significant impact on the recognition performance of the SAR-ATR system. In this experiment, we evaluated the model under standard operation conditions (SOCs), where the training and testing images were acquired under similar configurations, depression angles, etc. After the meta-learning, real images with depression angles of 17° and 15° were used to update and test the model, respectively. Our model was compared with several reference methods using different amounts of training data. Note that the term “training data” used in all the experiments refers to real SAR data to facilitate comparison with reference methods.

**Figure 5:**
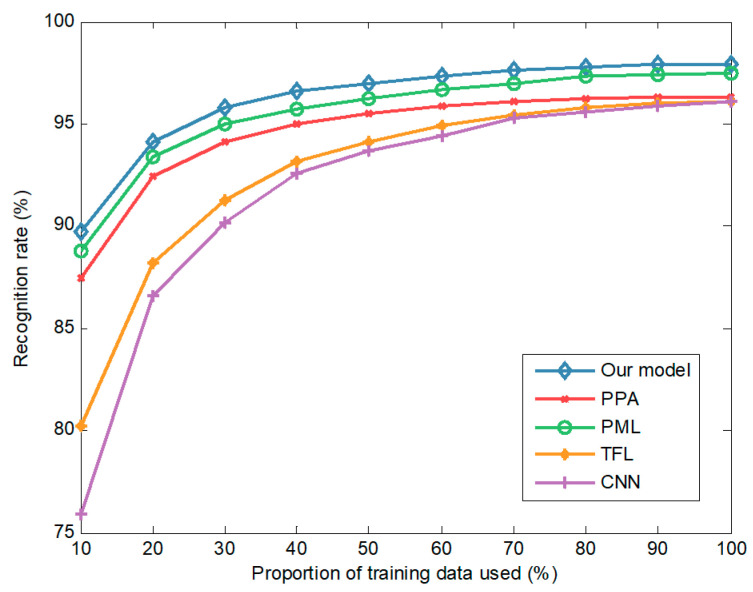
The recognition rates obtained from different amounts of training data.

Figure 5 shows that the recognition rates of all methods (our model, PML, PPA, TFL, and CNN) rise rapidly with increasing data, and then saturate when reaching a certain amount of training data. Our model achieves a recognition rate of more than 95% with only 30% of the data, indicating that it has excellent data use efficiency. When 10% of the training data are used, the recognition rate of the model is 89.7%, compared with 88.7% for PML, 87.4% for PPA, 80.2% for TFL, and 75.9% for CNN. In recognition tasks with small data, our model outperforms other methods by large margins. With more data, the recognition performance of all methods improves, and our model consistently achieves the best performance. When using 100% training data, the recognition rate for our model is 97.9%, which is 0.3%, 1.5%, 1.8%, and 1.8% better than those of the competitors, PML, PPA, TFL, and CNN, respectively. CNN performs the worst because it is trained only on the real data. By transferring knowledge from simulated to real data, the recognition rate of TFL is higher than that of CNN. Methods that use the meta-learning framework (i.e., our model, PML, and PPA) are superior to TFL, especially when training data are scarce. By introducing posterior distributions over parameters, our model can deal with the uncertainty caused by small data and hence perform well in small data scenarios.

The model was also compared with deep transferred atrous-inception synthetic aperture radar network (TAI-SARNET), TAI-SARNET with transfer learning (TAI-SARNET-TF), and MobileNet. These methods are lightweight network architectures that can be used for recognition in small-data scenarios. TAI-SARNET-TF1 transfers prior knowledge from optical data, TAI-SARNET-TF2 transfers prior knowledge from SAR data and TAI-SARNET-TF3 transfers knowledge from mixed data. Table 5 summarizes the recognition results with small sample sizes. The results of MobileNet were obtained from our implementation, while the results of TAI-SARNET and TAI-SARNET-TF were from [39]. Our model performs better than the competitors in small-data scenarios. When the proportion of training data is 1/2, the recognition rate of our model is 97.0%, which is 3.8%, 2.7%, 0.9%, 3.4%, and 5.5% higher than those of the competitors. When the proportion decreases to 1/32, our model surpasses the competitors by large margins.

**Figure 6:**
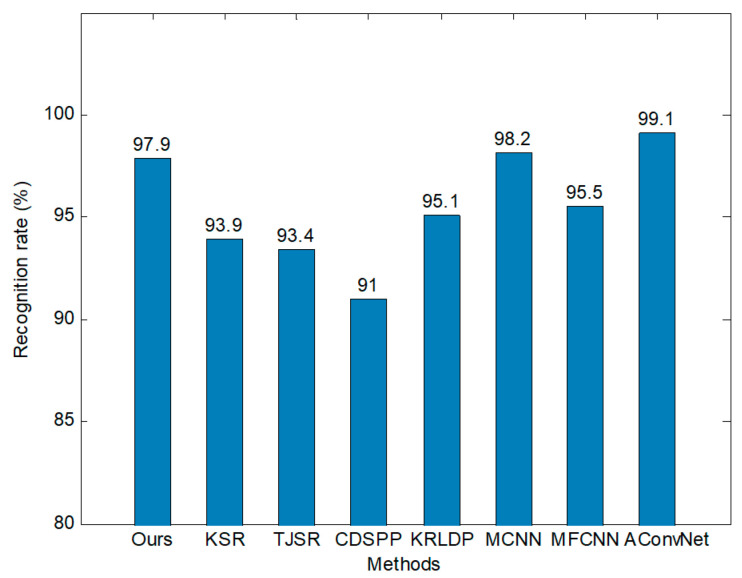
Comparison of different methods under the standard operation conditions (SOCs) test.

Finally, we compared the model with several SAR-ATR methods proposed in recent years. The recognition results in Figure 6 were obtained with 100% training data. The recognition rate of the model is marginally lower than those of all-convolutional network (A-ConvNet) and micro convolutional neural network (MCNN), but it is still higher than those of most reference methods. Although our model focuses on recognition tasks in small-data scenarios, it can realize performance improvement with more training data and achieves excellent results.

### 3.5. Results under Depression Angle Variations

**Figure 7:**
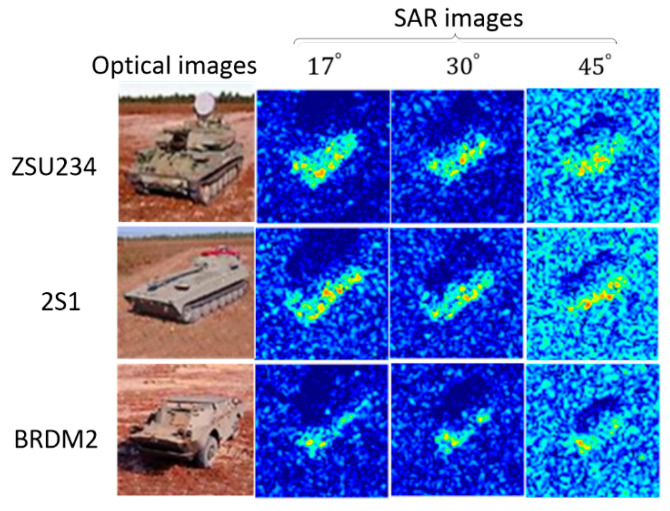
Illustration of target images at different depression angles. All targets have an azimuth angle of 45°.

In this test, the depression angles of training and testing images are markedly different, which is one of the extended operation conditions (EOCs). Figure 7 compares the target images at various depression angles. When the depression angles are not significantly different (17° versus 30°), the target shapes are similar, with only slight differences in scattering centers. When the depression angles differ noticeably (17° versus 45°), the target shape, scattering pattern, and even the speckle noise of the two images are different. Following the referenced methods, images of three targets (2S1, BRDM2, and ZSU234) were selected to evaluate the model. As shown in Table 6, the training set contains 890 images collected at a depression angle of 17°, and the test set contains 1778 images at depression angles of 30° and 45°.

**Figure 8:**
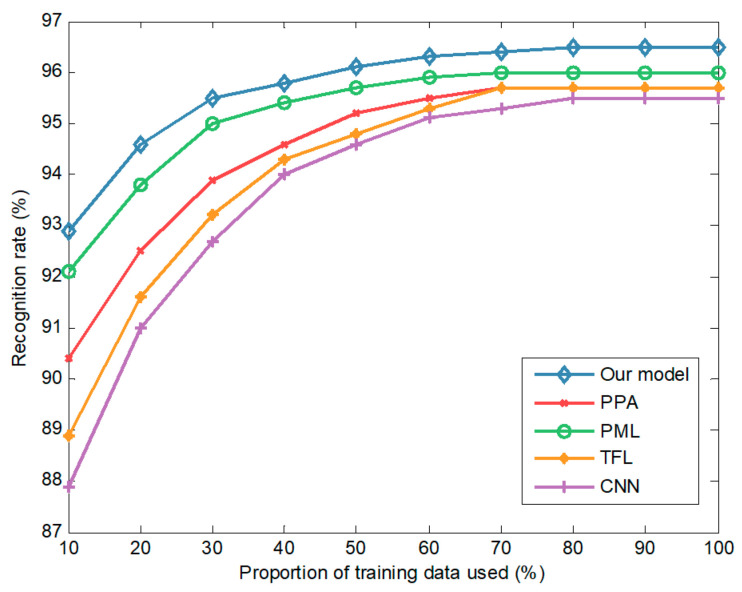
The recognition rates of different methods at a depression angle of 30°.

We compared the recognition rates of five methods at a depression angle of 30°, where training and testing images are slightly different. Figure 8 plots the recognition rates under different proportions of training data. When 10% of the training data is used, the recognition rates of the model, PML, PPA, TFL, and CNN are 92.9%, 92.1%, 90.4%, 89.0%, and 87.9%, respectively. Using the complete training data, the recognition rates of the five methods increase to 96.5%, 96.0%, 95.7%, 95.7%, and 95.5%, respectively. The recognition rates increase with the amount of training data, and our model is always better than the four competitors.

**Figure 9:**
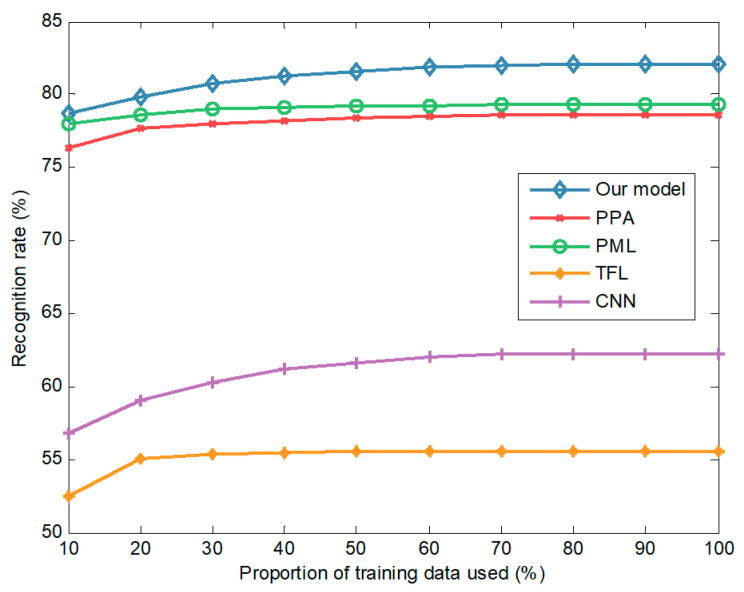
The recognition rates of different methods at a depression angle of 45°.

As shown in Figure 9, at a depression angle of 45°, the recognition performance of all methods deteriorates dramatically. When the proportion of training data is 10%, the recognition rates of our model, PML, PPA, TFL, and CNN are 78.7%, 78.0%, 76.4%, 52.5%, and 56.8%, respectively. When the proportion increases to 100%, the recognition rates of the five methods increase to 82.1%, 79.3%, 78.6%, 55.6%, and 62.2%, respectively. A drastic change in the depression angle significantly modifies the target’s appearance and hence leads to a huge difference between training and testing images. This difference makes the recognition methods gain little from the training data, thereby degrading their recognition performance. Despite using simulated SAR data, TFL performs worse than CNN trained only by the real data. One possible explanation is that the method of fine-tuning network parameters, which is used by TFL, is not suitable for this scenario.

**Figure 10:**
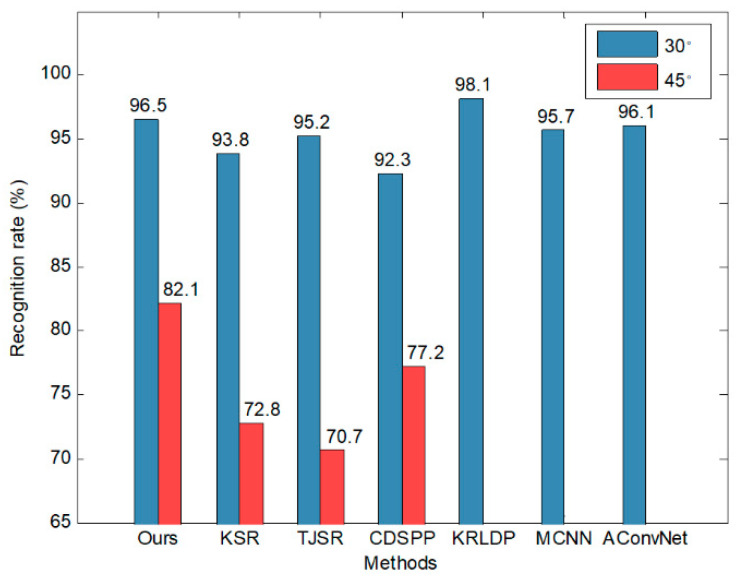
Comparison of different methods under the depression angle test. KRLDP, MCNN, and A-ConvNet only provide recognition results for a depression angle of 30°.

Finally, using the complete dataset, we compared the model with several reference SAR-ATR methods, and their recognition rates are plotted in Figure 10. Note that KRLDP, MCNN, and A-ConvNet only provide recognition results at a depression angle of 30°. When the depression angle of the test images (30°) is close to that of the training images (17°), all of the recognition rates are maintained at a high level of more than 90%. Our model is superior to all reference methods except KRLDP. When the testing depression angle increases to 45°, all of the recognition rates decrease sharply. Our model obtains a recognition rate of 82.1%, which surpasses the competing methods by large margins. This superior performance is due to the meta-learning and simulated data used in the model. Unlike reference methods only trained by real images with scarce depression angles, our model is meta-trained by a large number of simulated images with multiple depression angles. The learned angle-invariant global parameters make our model remain relatively robust under depression variations.

### 3.6. Results under Configuration Variations

**Figure 11:**
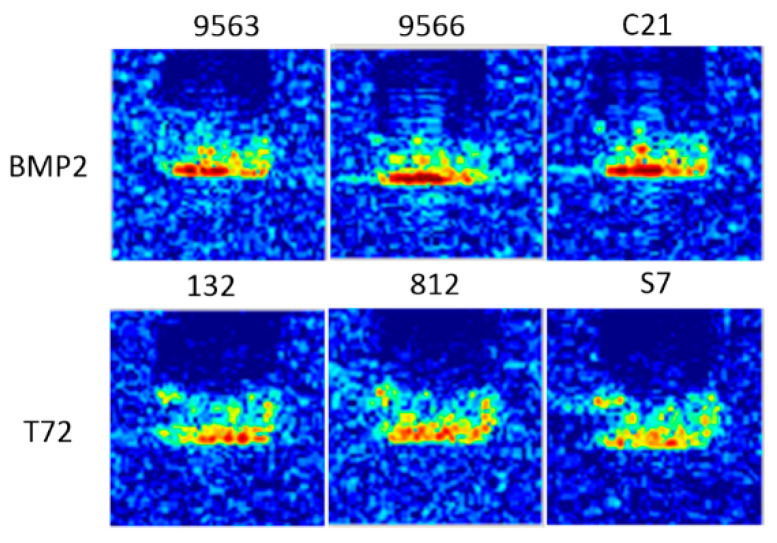
Illustration of target images in different configurations (titled by their serial numbers). All targets have an azimuth angle of 90° and a depression angle of 17°.

In this experiment, the targets used for training and testing have different configurations. Configuration refers to small appearance modifications, such as adding or removing fuel barrels, side skirts, and smoke grenade launchers to the targets. On the battlefield, targets of the same type but with different configurations should be classified into the same class. This is a challenging task because when two targets are just different in configurations, they have similar scattering characteristics. As shown in Figure 11, target images in three configurations are generally similar except for a slight difference in position and intensity of scattering centers. We trained and tested the model with four ground targets, in which T72 and BMP2 have three configuration variants indicated by different serial numbers. For T72 and BMP2, targets with the serial numbers 132 and 9563 were used for training, while the remaining ones, with the serial numbers S7, 812, C21 and 9566, were used for testing. For T62 and BTR60, their training and testing images have the same configuration, but with different depression angles. Table 7 summarizes the serial numbers and numbers of images used in this test.

**Figure 12:**
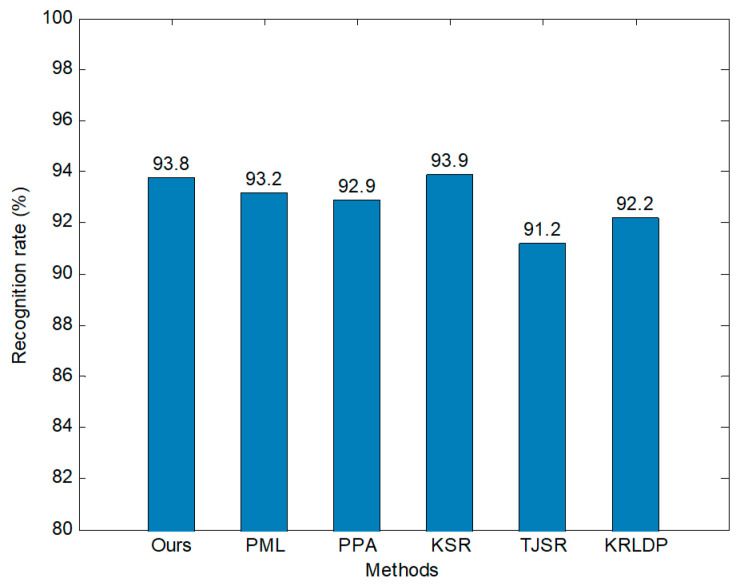
Comparison of different methods under the configuration test.

We compared the model with several reference methods and provide the results in Figure 12. For a fair comparison, all results were obtained from full training data. The recognition rate of our model is 93.8%, compared with 93.2% for PML, 92.9% for PPA, 93.9% for KSR, 91.2% for TJSR, and 92.2% for KRLDP. Our model performs better than most methods but slightly worse than KSR. Experimental results verify that the model can effectively solve recognition problems under configuration variations.

### 3.7. Evaluation of Model Calibration

With a considerable number of network parameters, CNN obtains high predictive accuracy, but it tends to be overconfident and is poorly calibrated. An overconfident CNN will assign a high confidence score (i.e., the softmax output at the end of the network) towards the wrong class for things it has not seen before. This makes it unsuitable for SAR-ATR systems that must not only provide predictions but also calibrated confidence measures. Only when the model is well-calibrated can we use the confidence score to judge the reliability of the recognition results. The results with low confidence can be passed to image analysts or supervisors for further inspection. Bayesian methods [31] offer a practical framework to address this shortcoming. Our model uses AVI to infer posterior distributions over task-specific parameters instead of calculating a point estimate of them. The posterior captures the uncertainty of these parameters and results in a well-calibrated model.

**Figure 13:**
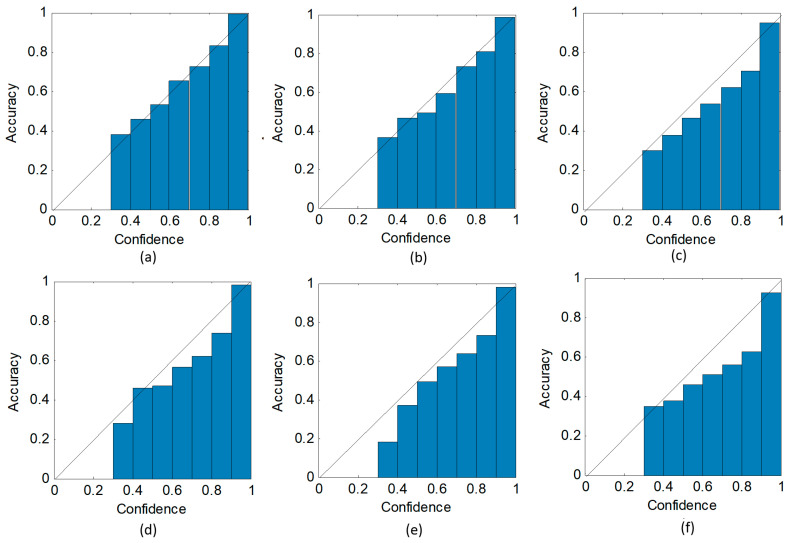
Reliability diagrams of (**a**) our model with 100% data, (**b**) our model with 50% data, (**c**) our model with 10% data, (**d**) PPA with 100% data. (**e**) PPA with 50% data, (**f**) PPA with 10% data.

First, we used reliability diagrams [41] to visually measure the model calibration. The reliability diagrams reflect the relationship between expected predictive accuracy and confidence score. The more aligned the bars and diagonals in reliability diagrams, the less the calibration error of the model. Figure 13 shows the reliability diagrams of our model and PPA for different amounts of training data. In each subplot of Figure 13, the expected accuracies are lower than the confidence scores, indicating a tendency towards overconfidence in both of the two methods. However, the gaps between the expected accuracies and confidence scores of our model are less than those of PPA. Our model provides a more calibrated confidence score by inferring posterior distributions over parameters.

Second, we used the expected calibration error (ECE) and the maximum calibration error (MCE) to quantify the model calibration. ECE and MCE represent the weighted average deviation and the worst-case deviation between the expected accuracy and confidence score of each bin, respectively. The lower the calibration error scores, the better the model calibration. Table 8 shows that both MCE and ECE of the model are always less than those of PPA under three different proportions of training data. It can also be observed that providing more training data can reduce MCE and ECE, thereby achieving better model calibration.

The above experimental results verify that our model performs better than PPA in model calibration. Our model uses posterior distributions to capture the randomness of task-specific parameters, while the PPA treats these parameters as deterministic values. Integrating over the posteriors will lead to a well-calibrated model.

### 3.8. Models with Different Network Structures

This section discusses the recognition results of models with different network structures. We designed eight different networks, summarized in Table 9. AVG represents an average-pooling operation. The difference between these networks lies in the number of convolutional kernels and whether the average pool is used at the end of the feature extractor. The last four rows of Table 9 show the implementation details of the weight predictor.

Table 10 compares the recognition results of different networks. All of the results were obtained under the SOC experimental setup. Networks using average pooling (B, D, F, H) are inferior to networks without average pooling (A, C, E, G), suggesting that average pooling degrades the recognition performance. The average pooling reduces the dimensions of feature vectors, which determine the number of hidden units in FC layers. The network capacity of the weight predictor is reduced with the decrease in the number of hidden units, thus degrading the recognition performance. Compared with the average-pooling, the number of convolutional kernels has less impact on recognition performance. Network C, used by our model, achieves the best result.

### 3.9. Recognition Results under Different Amounts of Simulated Data

In this section, we analyze the influence of simulated data on model recognition performance. We randomly selected 20%, 40%, 60%, 80%, and 100% images from the simulated dataset to construct small datasets. During meta-learning, these small simulated datasets are used to train the model. After the meta-learning, we used real SAR images at a depression angle of 17° to update the model and use real images at other depression angles (15°, 30°, 45°) to test it. Table 11 list the test results with different small simulated datasets. When using 100% simulated data, the recognition rates of the model at 15°, 30°, and 45° are 97.9%, 96.5%, and 82.1%, respectively. When the proportion drops to 20%, the recognition rates of the three test angles are reduced by 1.9%, 2.1%, and 5.3%, respectively. The recognition rate of the model decreases as the amount of simulated data decreases. The smaller the simulated data set, the smaller the number of azimuth angles, depression angles, and classes contained in it. The model cannot learn enough prior knowledge from such a small dataset, thus degrading its recognition performance. In order to further improve the recognition performance of the model, we should construct a complete simulated dataset. The dataset must contain a variety of target images, each of which covers complete azimuth and depression angles. Besides, the simulated images should have various ground clutters and speckle noises.

## 4. Conclusions

Recognition with small data has been a daunting problem in SAR-ATR because collecting sufficient real SAR data is difficult. In this paper, we propose a model incorporating meta-learning and AVI, which realizes the knowledge transfer from simulated data to real data. With meta-learning and simulated SAR data, our model can recognize novel targets using small amounts of real SAR data. Moreover, inferring the posterior distributions with AVI allows the model to provide calibrated confidence scores in addition to predictions. The results of extensive experiments verify that our model obtains state-of-the-art results, especially in the small-data scenario.

## Figures and Tables

**Table 1:** Description of the real SAR dataset.

Class	Depression	Number	Depression	Number
T72	17°	232	15°	196
BMP2	17°	233	15°	196
BTR60	17°	256	15°	195
BTR70	17°	233	15°	196
2S1	17°	299	15°	274
BRDM2	17°	298	15°	274
T62	17°	299	15°	273
D7	17°	299	15°	274
ZSU234	17°	299	15°	274
ZIL131	17°	299	15°	274

**Table 2:** Simulation parameters.

Parameters	Values
Center Frequency	9.6 G Hz
Resolution	0.3 m
Pixel Size	0.2 m
Bandwidth	0.5 G Hz
SAR Focusing	Spotlight
Weighting	Taylor, −35 db

**Table 3:** Description of the simulated SAR dataset.

Class	CAD Model	Number
Bulldozer#1	8020	504
Bulldozer#2	13,013	504
Bus#1	30,726	504
Bus#2	55,473	504
Car#1	Toyota	504
Car#2	Peugeot	504
Hummer#1	3663	504
Hummer#2	9657	504
Motorbike#1	3651	504
Motorbike#2	3972	504
Tank#1	65,047	504
Tank#2	86,347	504
Truck#1	2096	504
Truck#2	2107	504

**Table 4:** Reference methods that are studied in this paper.

Abbreviation	Full Name	Ref.
CNN	convolutional neural network	[15]
TFL	transfer learning	[25]
PPA	predicting parameters from activations	[33]
PML	probabilistic meta-learning	[32]
KSR	kernel sparse representation	[9]
TJSR	tri-task joint sparse representation	[10]
CDSPP	class-dependent structure preserving projection	[5]
KRLDP	kernel robust locality discriminant projection	[6]
MCNN	micro convolutional neural network	[18]
MFCNN	multiple feature-based convolutional neural network	[19]
A-ConvNet	all-convolutional network	[16]
TAI-SARNET	deep transferred atrous-inception synthetic aperture radar network	[39]
MobileNet	efficient convolutional neural networks for mobile vision applications	[40]

**Table 5:** Recognition results when using small sample sizes.

Methods	Recognition Rate Using Different Proportions of Training Data					
	1/32	1/16	1/8	1/4	1/3	1/2
Our model	70.1%	82.2%	89.6%	94.3%	95.7%	97.0%
TAI-SARNET	44.5%	67.0%	76.3%	88.7%	89.4%	93.2%
TAI-SARNET-TF1	56.7%	75.9%	84.9%	91.0%	92.8%	94.3%
TAI-SARNET-TF2	63.5%	80.1%	88.4%	94.1%	95.8%	96.1%
TAI-SARNET-TF3	60.0%	76.8%	82.2%	92.3%	93.3%	93.6%
MobileNet	29.6%	34.7%	45.6%	74.9%	86.2%	91.5%

**Table 6:** Dataset for the depression angle test.

Class	$\mathrm{Training}\;(17^{{}^{\circ}})$	$\mathrm{Test}\;(30^{{}^{\circ}})$	$\mathrm{Test}\;(45^{{}^{\circ}})$
2S1	299	298	299
BRDM2	288	287	288
ZSU234	303	303	303

**Table 7:** Dataset for the configuration test.

Class	$\mathrm{Training}(17^{{}^{\circ}})$		$\mathrm{Test}\left(15^{{}^{\circ}}\right)$	
	Serial Number	Number	Serial Number	Number
T72	132	232	S7, 812	386
T62	A51	299	A51	273
BMP2	9563	233	C21, 9566	392
BTR60	k10yt7532	256	k10yt7532	195

**Table 8:** Comparison of error scores using different amounts of training data.

Error Scores	10%	50%	100%
ECE of Our model	0.0573	0.0117	0.0082
ECE of PPA	0.0812	0.0267	0.0185
MCE of Our model	0.1506	0.0589	0.0435
MCE of PPA	0.2285	0.1375	0.1298

**Table 9:** Summary of different network structures.

A	B	C	D	E	F	G	H
Conv, 8 × 5 × 5		Conv, 16 × 5 × 5		Conv, 16 × 5 × 5		Conv, 32 × 5 × 5	
BN, ReLU, Dropout, Max-pooling							
Conv, 16 × 5 × 5		Conv, 32 × 5 × 5		Conv, 32 × 5 × 5		Conv, 64 × 5 × 5	
BN, ReLU, Dropout, Max-pooling							
Conv, 32 × 3 × 3		Conv, 64 × 3 × 3		Conv, 64 × 3 × 3		Conv, 128 × 3 × 3	
BN, ReLU, Dropout, Max-pooling							
Conv, 64 × 3 × 3		Conv, 64 × 3 × 3		Conv, 128 × 3 × 3		Conv, 256 × 3 × 3	
BN, ReLU, Dropout, Max-pooling							
--	AVG	--	AVG	--	AVG	--	AVG
Flattening							
FC, 1024	FC, 64	FC, 1024	FC, 64	FC, 2048	FC, 128	FC, 4096	FC, 256
FC, 1024	FC, 64	FC, 1024	FC, 64	FC, 2048	FC, 128	FC, 4096	FC, 256
FC, 2048	FC, 128	FC, 2048	FC, 128	FC, 4096	FC, 256	FC, 8192	FC, 512
FC, 1024	FC, 64	FC, 1024	FC, 64	FC, 2048	FC, 128	FC, 4096	FC, 256

**Table 10:** Recognition results of different networks.

Networks	A	B	C	D	E	F	G	H
Recognition results	97.3%	90.5%	97.9%	92.3%	97.5%	93.7%	97.0%	95.9%

**Table 11:** Recognition results with different small simulated datasets.

Test Depression Angles	Recognition Results				
	20%	40%	60%	80%	100%
15°	96.0%	96.8%	97.2%	97.6%	97.9%
30°	94.4%	95.3%	95.9%	96.2%	96.5%
45°	76.8%	78.1%	79.5%	81.7%	82.1%
